# Pharmacokinetic-guided versus weight-guided coagulation factor replacement in hemophilia patients undergoing arthroplasty: a retrospective real-world study on perioperative bleeding risk and economic outcomes

**DOI:** 10.3389/fmed.2026.1863312

**Published:** 2026-07-14

**Authors:** Shuxia Zhang, Yi Chen, Haojie Qin, Jing Chen, Chunrong Chen, Xuemei Wu, Min Chen, Jiajun Lin, Fenge Yang, Meijuan Huang

**Affiliations:** 1 Fujian Provincial Key Laboratory on Hematology, Fujian Medical University Union Hospital, Fujian Institute of Hematology, Fuzhou, China; 2 Department of Pharmacy, Fujian Medical University Union Hospital, Fuzhou, China; 3 Department of Orthopedic, Fujian Medical University Union Hospital, Fuzhou, China

**Keywords:** arthroplasty, coagulation factor replacement, hemophilia, perioperative care, pharmacokinetics

## Abstract

**Objective:**

This study aimed to compare the perioperative bleeding risk and economic outcomes of pharmacokinetic (PK)-guided versus weight-guided coagulation factor replacement regimens during the perioperative period for patients with hemophilia undergoing joint arthroplasty.

**Method:**

We conducted a retrospective analysis of clinical data from patients with hemophilia who underwent knee or hip joint arthroplasty at our center between 2013 and 2025.

**Results:**

Thirty-three male patients with a median age of 39 years were included. Twenty-five patients (75.8%) had severe hemophilia. Seventeen patients (51.5%) underwent total joint arthroplasty, and 16 (48.5%) underwent partial joint arthroplasty. Perioperative coagulation factor replacement was weight-guided in 22 patients (66.7%) and PK-guided in 11 patients (33.3%). Postoperative SF-6Dv2 scores indicated a significant improvement in quality of life compared to preoperative baselines. The PK-guided group demonstrated a significantly lower median hospitalization cost (138,232 RMB vs. 200,122 RMB, *p* = 0.0073) and lower median coagulation factor consumption (701.6 IU/kg vs. 892.9 IU/kg, *p* = 0.0304) compared to the weight-guided group. Median intraoperative blood loss and perioperative hemoglobin decrease were comparable between groups.

**Conclusion:**

For patients with hemophilia undergoing joint arthroplasty, a PK-guided coagulation factor infusion regimen significantly reduced factor consumption and overall medical costs, without a statistically significant increase in perioperative bleeding risk compared with the weight-guided approach. These exploratory findings support the clinical and economic advantages of PK-guided management in this surgical setting and warrant confirmation in larger, prospective studies.

## 1 Introduction

Hemophilia is one of the most common genetic bleeding disorders. Patients with hemophilia are prone to recurrent hemarthroses as the disease progresses, leading to hemophilic arthropathy (HA) (1, 2). This condition can ultimately result in joint deformity and functional loss, representing one of the primary severe complications that impair the quality of life for these patients (3, 4). For patients with end-stage hemophilic arthropathy, arthroplasty is currently the only effective treatment in clinical practice (5, 6). However, due to the inherent coagulation abnormalities in hemophilia patients, their perioperative bleeding risk is significantly higher than that of patients undergoing standard orthopedic surgery (7). Consequently, coagulation factor replacement therapy is a critical determinant of both surgical safety and therapeutic outcomes (8).

Traditionally, clotting factor replacement therapy has been dosed according to a patient’s body weight using a fixed formula (9). This approach, however, fails to adequately account for individual patient variables such as age, hepatic and renal function, and the clearance rate of the clotting factor, resulting in significant limitations in clinical practice (10). In recent years, pharmacokinetic (PK)-guided clotting factor replacement therapy has emerged as a key focus of clinical research in this field (11–13). However, most current studies concentrate on validating the efficacy of a single therapeutic strategy, with few direct comparative studies between different approaches for hemophilia patients undergoing arthroplasty (14, 15). The available evidence is largely derived from small-scale studies on non-arthroplasty procedures or from PK analyses based on healthy populations (16), which deviates considerably from real-world clinical scenarios.

In light of these considerations, this retrospective study aims to compare weight-guided and PK-guided replacement therapies in hemophilia patients undergoing arthroplasty, mainly focusing on coagulation factor consumption, bleeding risk, and medical costs. This study aims to provide practical guidance for the management of hemophilia patients undergoing arthroplasty.

## 2 Methods

### 2.1 Diagnosis of hemophilia and indications for arthroplasty

The diagnosis of hemophilia was established in accordance with the relevant criteria from the World Federation of Hemophilia (WFH) guideline for the Management of Hemophilia, based on a combination of clinical manifestations, coagulation function tests, and genetic testing results (17). The surgical indications for arthroplasty were also guided by WFH guideline for management of hemophilic arthropathy, which are applied to patients with severe joint deformity, intractable pain, and significant functional limitation.

### 2.2 Study design and patients included

This is a retrospective study that collected the clinical data of hemophilia patients who underwent orthopedic arthroplasty, including knee or hip replacement, at Fujian Medical University Union Hospital from January 2013 to June 2025. Due to the nature of clinical practice evolution, the PK-guided protocol was introduced at our center in 2020. Consequently, patients enrolled before 2020 were exclusively in the weight-guided group, while patients enrolled after 2020 were non-randomly assigned to either the PK-guided or weight-guided group. Distribution of surgery years for included patients was summarized in Supplementary Table 1. The selection of patients for treatment was based on a shared decision-making process between the physician and the patient after 2020, considering patient preference and clinical factors such as a history of unexplained bleeding or atypical response to previous factor replacement. The collected data included patient age, weight, perioperative clotting factor infusions, bleeding events and medical cost. Quality of life (QoL) was evaluated with the self-reported Short Form 6-dimensions version 2 (SF-6Dv2) instrument (18) at two time points: prior to surgery (baseline) and at the 12-month follow-up visit. This study was conducted in accordance with the principles of the Declaration of Helsinki, and ethical approval for the study was granted by the Ethics Committee of the Fujian Medical University Union Hospital, under the protocol number 2025KY129. Due to the retrospective design of the study, it was exempt from the requirement to obtain written informed consent from the patients for their participation.

### 2.3 Coagulation factor replacement protocols

The replacement therapy of choice is either recombinant clotting factor (VIII/IX) or virus-inactivated plasma-derived clotting factor (VIII/IX). The target factor levels for this type of surgery and the calculation of the weight-guided dosing were determined in accordance with the WFH guideline. The details of the treatment protocols are as follows:

PK-guided group: following at least two washout periods, all patients received a single intravenous injection of clotting factor (50 IU/kg for Hemophilia A, 80 IU/kg for Hemophilia B). Venous blood samples were collected at seven time points after the infusion was complete (0 h, 0.5 h, 3 h, 8 h, 24 h, 31 h, and 48 h). Based on the clotting factor activity levels at these seven points, along with patient weight and baseline levels, individual pharmacokinetic (PK) parameters (including peak concentration, trough concentration, clearance, and half-life) were calculated for each patient. These parameters, integrated with a population PK model, were used to precisely generate individual factor concentration-time curves and to formulate a personalized perioperative clotting factor replacement regimen for each patient. For the detailed model construction process, please refer to our previously published article (19).

Weight-guided group: dosing for clotting factor replacement therapy was calculated solely based on the patient’s body weight. This approach did not account for individual variations in factor metabolism, blood type, or von Willebrand factor (VWF) concentration, and a fixed dose and administration interval were used for perioperative replacement therapy.

### 2.4 Hemorrhage monitoring

Considering that the bleeding risk in hemophilia patients undergoing joint replacement is primarily determined by the severity of the clotting factor deficiency and the type of surgery, this study stratified patients into subgroups based on these two factors. Intraoperative blood loss was measured by the surgical team throughout the surgical procedure. Perioperative hemoglobin drop was measured by the magnitude of hemoglobin drop (ΔHb), defined as the difference between a patient’s preoperative and postoperative Hb levels. Specifically, ΔHb was calculated as the preoperative Hb value (from the most recent complete blood count) minus the lowest postoperative Hb value within 24–48 h. For patients receiving red blood cell (RBC) transfusions, a correction was applied: one unit of packed RBCs was estimated to increase Hb by approximately 10 g/L. Corrected ΔHb (g/L) = Preop Hb - Postop Hb + (Number of RBC units transfused × 10).

### 2.5 Statistical parameters and analysis

Length of stay (LOS) was defined as the total number of calendar days from the date of admission to the date of discharge. Total hospitalization cost was defined as the sum of all medical expenses incurred during the inpatient period, including surgical fees, clotting factor drug costs, and other related expenditures, measured in Chinese Yuan (RMB). Clotting factor consumption was defined as the total amount of all clotting factors used throughout the entire perioperative period, measured in International Units (IU).

Differences among patient subgroups were statistically evaluated using the chi-square test for categorical variables, the t-test for normally distributed continuous data, or nonparametric tests for data not meeting the assumptions of parametric tests. All statistical analyses and graphical representations were conducted using GraphPad Prism 9.5 (GraphPad Software, San Diego, CA, USA). A two-sided *p*-value of < 0.05 was considered statistically significant.

## 3 Results

### 3.1 Patients characteristics

A total of 33 male patients were enrolled in this study, with a median age of 39 years (range, 22–61 years). The median body weight was 68 kg (range, 45–87 kg), and the median body mass index (BMI) was 22.7 kg/m^2^ (range, 15.6–29.3 kg/m^2^). Of the patients, 29 (87.9%) had hemophilia A and 4 (12.1%) had hemophilia B. A total of 25 patients (75.8%) were classified as having severe hemophilia, while 8 patients (24.2%) had non-severe hemophilia. Only one patient exhibited a borderline inhibitor level of 0.6 BU/ml. Seventeen patients (51.5%) underwent total knee or hip arthroplasty, and 16 patients (48.5%) underwent partial knee or hip arthroplasty. During the perioperative period, 22 patients (66.7%) received weight-guided coagulation factor infusion, while 11 patients (33.3%) received PK-guided infusion. There were no significant differences between the two groups in terms of age, weight, hemophilia type and severity distribution, and surgical procedures. The detailed baseline characteristics of the two groups are presented in Table 1.

**Table 1:** Baseline characteristics of patients included in this study (*N* = 33).

Parameters	Total (*N* = 33)	PK-guided group (*n* = 11)	Weight-guided group (*n* = 22)	*p*-value
Age, year (range)	39 (22–61)	43 (22–61)	38 (23–60)	0.3475
Weight, kg (range)	68 (45–87)	60 (45–75)	69 (49–87)	0.2480
BMI, kg/m^2^ (range)	22.7 (15.6–29.3)	21.1 (15.6–29.3)	23.5 (16.9–27.2)	0.4333
Hemophilia type, *n* (%)				
Type A	29 (87.9)	10 (90.91)	19 (86.36)	>0.9999
Type B	4 (12.1)	1 (9.09)	3 (13.64)	
Disease severity 1, *n* (%)				
Non-Severe, *n* (%)	8 (24.2)	2 (18.2)	6 (27.3)	0.6870
Severe, *n* (%)	25 (75.8)	9 (81.8)	16 (72.7)	
Surgical procedure, *n* (%)				
Total knee or hip arthroplasty	17 (51.5)	7 (63.6)	10 (45.4)	0.4646
Partial knee or hip arthroplasty	16 (48.5)	4 (36.4)	12 (54.5)	

1. Severe hemophilia is defined as having a clotting factor activity level below 1%. The non-severe category, which includes moderate and mild hemophilia, is defined by factor activity levels between 1 and 40%.

### 3.2 Quality of life

A comparison of the SF-6D dimension scores before and after surgery is presented in Table 2. All six dimensions—Physical Functioning (PF), Social Functioning (SF), Role Limitations (RL), Bodily Pain (BP), Mental Health (MH), and Vitality (VT)—showed a statistically significant decrease from preoperative baseline values at the 12-month assessment (all *p* < 0.0001). Further analysis revealed that the differences in QoL between preoperative and postoperative assessments were not only statistically significant but also clinically meaningful. Postoperative Cohen’s d values for PF (2.37), SF (2.35), RL (2.09), BP (2.19), MH (1.77), and VT (1.26) all demonstrated substantial effect sizes, indicating significant improvements in patients’ postoperative QoL.

**Table 2:** Comparison of SF-6Dv2 dimension scores preoperatively and at 12 months postoperatively.

Dimension	Preoperative score	Postoperative score	*P*-value	Cohen’s d
Physical functioning (PF)	81.52 ± 14.39	48.79 ± 13.17	< 0.0001	2.37
Social functioning (SF)	77.27 ± 15.87	46.67 ± 9.24	< 0.0001	2.35
Role limitations (RL)	79.70 ± 16.30	47.88 ± 14.09	< 0.0001	2.09
Bodily pain (BP)	67.27 ± 17.01	37.58 ± 8.67	< 0.0001	2.19
Mental health (MH)	72.42 ± 18.38	43.94 ± 13.21	< 0.0001	1.77
Vitality (VT)	66.36 ± 18.34	45.15 ± 15.23	< 0.0001	1.26

### 3.3 Length of stay and costs

**Figure 1:**
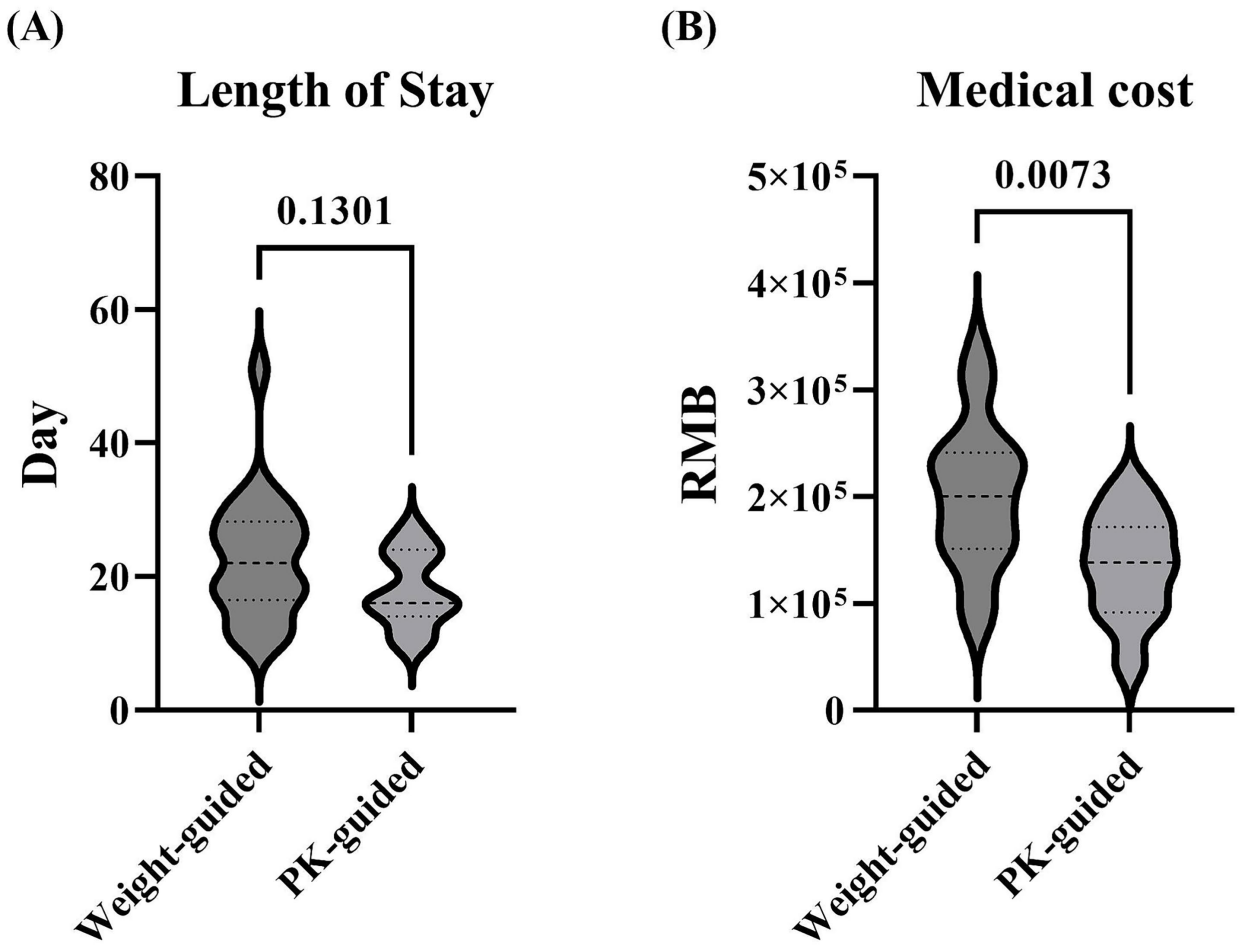
Length of hospital stay and total hospitalization costs. **(A)** Length of hospital stay: The median length of stay was 16 days (range, 10–27 days) in the PK-guided group and 22 days (range, 10–51 days) in the weight-guided group (*p* = 0.1301). **(B)** Total hospitalization costs: The median total cost was 138,232 RMB (range, 138,232–138,232 RMB) in the PK-guided group and 200,122 RMB (range, 78,326–339,813 RMB) in the weight-guided group (*p* = 0.0073).

As shown in Figure 1A, the median LOS was 16 days (range, 10–27 days) in the PK-guided group, compared to 22 days (range, 10–51 days) in the weight-guided group. The LOS for the PK-guided group was shorter than that of the weight-guided group, but the difference did not reach statistical significance (*p* = 0.1301). Regarding hospitalization costs, the median total cost for the PK-guided group (138,232 RMB; range, 43,758–209,344 RMB) was significantly lower than that for the weight-guided group (200,122 RMB; range, 78,326–339,813 RMB), with a *p*-value of 0.0073 (Figure 1B).

### 3.4 Perioperative factor consumption

**Figure 2:**
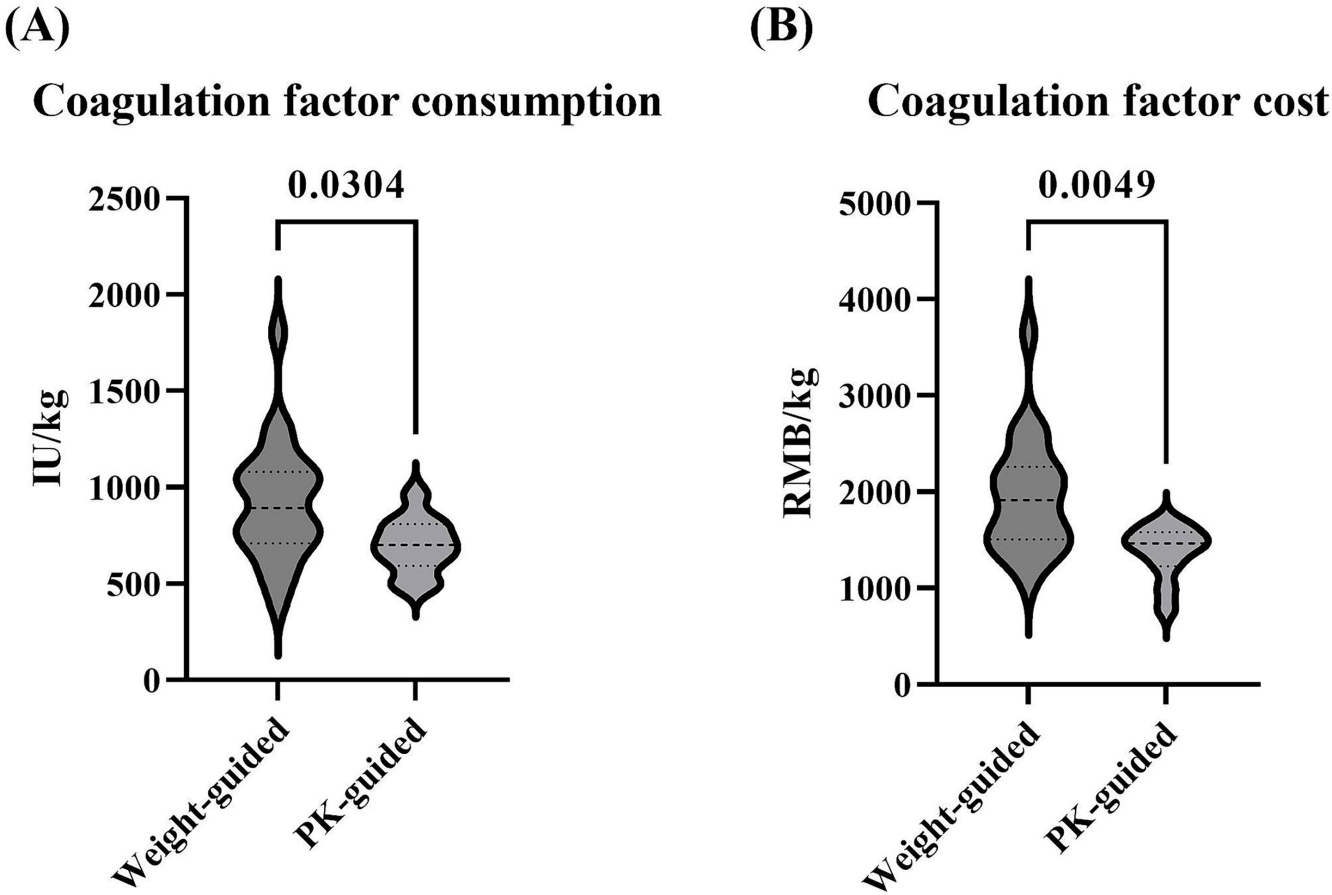
Perioperative clotting factor consumption. **(A)** The median consumption was 701.6 IU/kg (range, 490.9–963.3 IU/kg) in the PK-guided group and 892.9 IU/kg (range, 400.0–1803.0 IU/kg) in the weight-guided group (*p* = 0.0304). **(B)** The median coagulation factor cost in the PK-guided group was 1,464 RMB/kg (range: 784–1,686 RMB/kg), which was significantly lower than that in the weight-guided group (1,914 RMB/kg; range: 1,072–3,647 RMB/kg; *p* = 0.0049).

In this study, clotting factor consumption was statistically analyzed as the amount consumed per kilogram of body weight. As it was shown in Figure 2, the median consumption in the PK-guided group was 701.6 IU/kg (range, 490.9–963.3 IU/kg), which was significantly lower than that in the weight-guided group at 892.9 IU/kg (range, 400.0–1803.0 IU/kg), *p* = 0.0304. The median coagulation factor cost in the PK-guided group was 1,464 RMB/kg (range: 784–1,686 RMB/kg), which was significantly lower than that in the weight-guided group (1,914 RMB/kg; range: 1,072–3,647 RMB/kg; *p* = 0.0049). Notably, 100% of patients (11/11) in the PK-guided group actually achieved the recommended target clotting factor activity levels postoperatively, compared to 77.3% (17/22) in the weight-guided group (*p* = 0.1435).

### 3.5 Perioperative bleeding severity

In the PK-guided group, 2 of 11 patients received blood transfusions, compared with 5 of 22 patients in the weight-guided group (*p* > 0.9999). Similarly, one patient in each group developed a postoperative wound hematoma. There were no significant differences between the two groups in the incidence of blood transfusions or postoperative hematomas (*p* > 0.9999). At presented at Table 3, the median intraoperative blood loss during joint replacement surgery was 500 mL, with a range of 50–1,600 mL. The severity of hemophilia and the type of joint replacement procedure performed do not significantly influence intraoperative blood loss. The median intraoperative blood loss was lower in the PK-guided group (350 mL; range, 50–1,000 mL) compared with the weight-guided group (550 mL; range, 100–1,600 mL), though this difference did not reach statistical significance (*p* = 0.1485). The median perioperative hemoglobin decrease was 37 g/L, with a range of 11–96 g/L, and there were no differences in terms of disease severity, surgical procedure, and treatment protocol.

**Table 3:** Intraoperative Blood Loss and Perioperative Hemoglobin Drop.

Parameters	Intraoperative blood loss median (range), mL	*P*-value	Perioperative ΔHb median (range), g/L	*p*-value
Total, *N* = 33	500 (50–1,600)		37 (11–96)	
Disease severity				
Severe hemophilia, *N* = 25	500 (50–1,000)	0.3161	37 (11–76)	0.3578
Non-severe hemophilia, *N* = 8	550 (50–1,600)		39 (19–96)	
Surgical procedure				
Total KR/HR, *N* = 17	500 (50–1,600)	0.5531	37 (11–96)	0.7638
Partial KR/HR, *N* = 16	550 (50–1,200)		38 (19–76)	
Treatment protocols				
PK-guided group, *N* = 11	300 (50–1,000)	0.1485	33 (11–82)	0.3055
Weight-guided group, *N* = 22	550 (100–1,600)		40 (23–96)	

### 3.6 Sensitivity analysis adjusting for calendar era

Given the extended study period (2013–2025) and the implementation of PK-guided therapy only after 2020, temporal variations in factor pricing, surgical techniques, perioperative management, and LOS may have confounded the comparison. To minimize the potential influence of calendar era, we performed a subgroup analysis restricted to patients treated after 2020. In this restricted cohort, 11 patients received PK-guided therapy and 13 patients received weight-guided therapy. The median coagulation factor consumption was 701.6 IU/kg (range, 490.9–963.3 IU/kg) in the PK-guided group versus 821.3 IU/kg (range: 400.0–1071.0 IU/kg) in the weight-guided group (*p* = 0.0647). The median total hospitalization cost was 138,232 RMB (range: 43,758–209,344 RMB) in the PK-guided group and 167,639 RMB (range: 78,326–244,267 RMB) in the weight-guided group (*p* = 0.1432). The median length of stay was 16 days (range: 10–27 days) in the PK-guided group and 18 days (range: 8–51 days) in the weight-guided group (*p* = 0.6191). Although numerical differences favoring the PK-guided group persisted, none reached statistical significance, likely due to the limited sample size.

## 4 Discussion

This retrospective study provides real-world evidence comparing the efficacy, safety, and economic impact of PK-guided versus weight-guided coagulation factor replacement therapy in patients with hemophilia undergoing joint arthroplasty. The principal findings demonstrate that PK-guided management significantly reduces factor consumption and overall hospitalization costs, while maintaining perioperative safety that did not differ significantly between groups and achieving superior postoperative factor activity target attainment, without compromising the substantial improvement in QoL afforded by surgery.

The observed 21.4% reduction in median factor consumption per kilogram (701.6 vs. 892.9 IU/kg) in the PK-guided group aligns with the fundamental premise of personalized dosing. Weight-based protocols, while pragmatic, often overlook individual variations in factor half-life, recovery, and clearance, potentially leading to supra-therapeutic dosing to ensure hemostatic coverage (20). In contrast, PK-guided dosing tailors the regimen to individual patient pharmacokinetics, aiming to maintain factor activity above the therapeutic threshold more precisely, thereby minimizing waste (21, 22). This mechanistic efficiency is further corroborated by our finding that 100% of patients in the PK-guided group achieved postoperative target factor activity levels, compared to 77.3% in the weight-guided group, although this difference was not statistically significant, likely due to the limited sample size. This trend suggests that PK guidance may offer more consistent and reliable hemostatic protection.

The significant reduction in total hospitalization costs (approximately 31%, 138,232 vs. 200,122 RMB) in the PK-guided group is a critical outcome, directly attributable to the decreased consumption of expensive clotting factor concentrates. While the shorter median LOS in the PK group (16 vs. 22 days) did not reach statistical significance, it contributed to the overall cost savings and suggests a potential trend toward more efficient recovery. The dramatic improvement in QoL across all SF-6D domains postoperatively, with large effect sizes, underscores the transformative benefit of joint replacement in this population. Importantly, this benefit was achieved equally in both groups, confirming that the economic and dosing efficiencies of PK guidance do not come at the expense of the primary surgical goal—restoring function and alleviating pain.

The comparable safety profiles between the two strategies are reassuring. There were no significant differences in intraoperative blood loss or perioperative hemoglobin decrease, suggesting that the reduced factor dosing in the PK group was not associated with an increased risk of bleeding in this cohort. This finding is consistent with the principle that PK guidance aims for optimal, not minimal, dosing, ensuring hemostasis while avoiding excess.

Several limitations of this study must be acknowledged. Its retrospective, single-center design and small sample size limit the statistical power to detect differences in secondary outcomes, such as LOS and target attainment rates, and increase the risk of confounding. Most importantly, the study is substantially underpowered for safety outcomes. Therefore, the findings of this study should be considered exploratory, and the results regarding bleeding events should be interpreted with caution. The non-randomized assignment to treatment groups, though balanced in baseline characteristics, may introduce selection bias. Furthermore, the frequency of postoperative factor activity monitoring did not adhere strictly to ideal PK sampling schedules, which may have limited the full dynamic optimization of dosing in the PK group.

Despite these limitations, our findings contribute valuable “real-world” data supporting the clinical and economic advantages of PK-guided therapy in a high-risk surgical setting. Future research should focus on prospective, multicenter, randomized controlled trials with larger cohorts to confirm these benefits and establish higher-level evidence. Such studies should incorporate more intensive perioperative monitoring to fully elucidate the dynamic dosing adjustments enabled by PK models and include long-term follow-up to assess the impact on joint health, prosthesis survival, and sustained QoL improvements. Investigating the integration of PK guidance with emerging point-of-care monitoring technologies could further refine and simplify personalized perioperative management for patients with hemophilia.

In conclusion, this analysis suggests that PK-guided coagulation factor replacement is a viable and advantageous strategy for the perioperative management of hemophilia patients undergoing major orthopedic surgery. It offers a path to significant cost savings and more efficient resource utilization while maintaining safety and therapeutic efficacy, supporting its broader adoption in clinical practice.

## Data Availability

The original contributions presented in the study are included in the article/Supplementary material, further inquiries can be directed to the corresponding authors.
