# Supplementary material for: Pharmacokinetic-guided versus weight-guided coagulation factor replacement in hemophilia patients undergoing arthroplasty: a retrospective real-world study on perioperative bleeding risk and economic outcomes
Source: Front Med (Lausanne). 2026 Jul 14;13:1863312. doi: 10.3389/fmed.2026.1863312 (PMC13407202; doi:10.3389/fmed.2026.1863312)
Supplement: Supplementary file 1 [file Table_1.DOCX]

| Supplementary Table 1. Distribution of Surgery Years for Included Patients | | |
| --- | --- | --- |
| Year | Weight-Guided | PK-Guided |
| 2013 | 1 | 0 |
| 2014 | 2 | 0 |
| 2015 | 3 | 0 |
| 2016 | 1 | 0 |
| 2017 | 2 | 0 |
| 2018 | 0 | 0 |
| 2019 | 0 | 0 |
| 2020 | 1 | 1 |
| 2021 | 3 | 0 |
| 2022 | 2 | 1 |
| 2023 | 2 | 3 |
| 2024 | 4 | 5 |
| 2025 | 1 | 1 |
